# Real-world cost-effectiveness of nirmatrelvir-ritonavir as treatment for SARS-CoV-2 infection in the Belgian setting with omicron variant

**DOI:** 10.3389/fpubh.2024.1432821

**Published:** 2025-02-03

**Authors:** Sophie Marbaix, Steven Simoens, Philippe Clevenbergh, Pascal Van Bleyenbergh, Keliane Liberman, Dimitri Dehenau

**Affiliations:** ^1^Health Economics, SNB Management, Soignies, Belgium; ^2^Faculty of Medicine and Pharmacy, Research Institute for Health Sciences and Technology, University of Mons–UMONS, Mons, Belgium; ^3^Department of Pharmaceutical and Pharmacological Sciences, KU Leuven, Leuven, Belgium; ^4^Head of Infectious Diseases Department, CHU Brugmann, Brussels, Belgium; ^5^Head of Pneumology Department, KU Leuven, Leuven, Belgium; ^6^Medical Department, Pfizer, Brussels, Belgium; ^7^Access and Value Department, Pfizer, Brussels, Belgium

**Keywords:** cost-utility, SARS-CoV-2, antiviral treatment, Belgium, high-risk persons

## Abstract

**Background:**

Nirmatrelvir-ritonavir is an oral treatment for SARS-CoV-2 infection in patients who are at high risk of developing severe COVID-19 disease. This antiviral has proven to significantly reduce the risk of hospitalization and death compared to no anti-SARS-CoV-2 treatment in this target population. This paper aims to assess the cost-effectiveness of nirmatrelvir-ritonavir in Belgium using real-world evidence.

**Methods:**

A static decision tree model was developed to capture the health progression of patients infected with the SARS-CoV-2 virus. Outcomes were expressed in Quality Adjusted-Life Years (QALYs), hospitalizations, Intensive Care Unit (ICU) admissions, deaths and Long Covid cases, derived from epidemiological data over the first full year of the Omicron variant’s circulation (2022). Costs were calculated for the year 2023 from the healthcare payer’s perspective. Extensive sensitivity analyses were conducted to test the robustness of the cost-effectiveness results.

**Results:**

In a cohort of 1,000 patients, treatment with nirmatrelvir-ritonavir is projected to save 95 QALYs and €82,658 compared to no anti-SARS-CoV-2 treatment over a lifetime horizon. These savings primarily stem from the reduction in hospitalizations among vulnerable patients who typically require a longer recovery time. The analysis also indicates 5 fewer ICU admissions and 8 fewer premature deaths per 1,000 infected patients.

**Conclusion:**

In the context of Omicron SARS-CoV-2 infection, administering nirmatrelvir-ritonavir to patients at high risk of severe disease improves health outcomes and reduces costs. Nirmatrelvir-ritonavir is 100% likely to be cost-effective at a willingness to pay of €2,000 per QALY.

## Introduction

In December 2019, the world faced the emergence of the Severe Acute Respiratory Syndrome Coronavirus 2 (SARS-CoV-2). Its rapid and unprecedented global spread led the World Health Organization (WHO) to declare a pandemic in March 2020. The Coronavirus Disease 2019 (COVID-19) caused by this new virus is characterized by a wide range of symptoms, with most common being cough, fever, fatigue and difficult breathing (1, 2). The severity of the disease varies, ranging from asymptomatic cases to lethal infections. Over time, the transmissibility and severity of the virus have evolved. However, COVID-19 has had a significant impact on the Belgian population, particularly among the older adults (3). More than 90% of the COVID-19 burden was due to premature mortality, with the remainder attributed to long-lasting post-acute symptoms (3). Epidemiological data have been closely monitored by Sciensano, the Belgian Institute of health statistics. From the start of the pandemic up to December 2021 (during the alpha and delta variant period), Sciensano registered an average of 4,406 hospitalizations per month, indicating that 4.7% of the confirmed COVID-19 cases in the Belgian population required hospitalization (4). During the first year of Omicron variant (2022), the average number of monthly hospitalizations slightly decreased to 4,043. However, due to the higher transmissibility and incidence associated with the Omicron variant, the percentage of hospitalization decreased to 1.9% of the confirmed cases.

The risk of severe COVID-19 disease and death escalates with age and the presence of certain underlying conditions (5–8). This increased risk is also applicable to the most recent known variant of SARS-CoV-2, Omicron. Throughout 2022, the risk of hospitalization for infected adults aged 65 years and older was estimated at 12.2%. During their hospital stay, these older adults faced a 4.9% risk of being admitted to the intensive care unit. The risk of death remained at 6.4% of all hospitalized older adult patients (4, 9). Patients who suffered from at least one severe condition as defined by the KCE task force (10) and were not vaccinated experienced similar consequences with Omicron variant of SARS-CoV-2 infection: it was estimated that 11.5% of these patients were hospitalized, 6.4% of these hospitalized patients were admitted in ICU and 5.9% died in the hospital consequently to the infection (9).

The 3 years of the COVID-19 pandemic has severely disrupted people’s physical and mental health, with some patients experiencing long-term effects known as Long Covid symptoms (11, 12). Long Covid has impacted patients’ quality of life, social and professional lives. The worldwide economy has also been heavily undermined by the COVID-19 pandemic (13, 14). Although the pandemic is over, SARS-CoV-2 and its new variants are still circulating, it has become endemic. Therefore, there is a need to appropriately address a potential resurgence of new variants of this virus or other coronaviruses. Vaccines (15) and/or specific antiviral treatments are complementary interventions that can optimally protect vulnerable patients.

COVID-19 vaccines have some limitations: the emergence of new variants requires vaccine adaptations and boosters to maintain strong protection (16, 17), the efficacy of vaccination wanes over time (18), the immune response in the older adult population and clinically fragile patients is lower (19, 20), there are logistical issues for administration and some individuals are hesitant to get vaccinated.

Nirmatrelvir-ritonavir, due to its specific inhibition of the viral proteases (M^pro^), is a pan-coronavirus inhibitor. It has proven to be effective against all known SARS-CoV-2 variants and strains and is expected to maintain activity against new variants (21–24).

Paxlovid® (nirmatrelvir-ritonavir) has been studied in high-risk, non-hospitalized adults infected by SARS-CoV-2 and has been recommended in the European Union for the treatment of COVID-19 in adults who do not require supplemental oxygen and who are at increased risk for progressing to severe COVID-19. When administered within the first 5 days of symptoms, nirmatrelvir-ritonavir has shown to reduce the severity of COVID-19, including a decrease of risk of hospitalization and of death (25). These clinical outcomes of nirmatrelvir-ritonavir have been confirmed in real-world settings, though not specifically to the Belgian context: an observational study conducted in the United States reported an 80% efficacy of nirmatrelvir-ritonavir against hospitalization and/or death in older patients and patients with underlying conditions (more specifically cardiovascular and respiratory conditions), whether they were vaccinated or not (26). Ongoing surveillance and *in vitro* data also indicated a low potential for nirmatrelvir-ritonavir resistance, suggesting sustained treatment efficacy with continued widespread use (27). Preventing severe COVID-19 also results in lower risk of Long Covid symptoms. Xie et al. reported that the administration of nirmatrelvir-ritonavir within the first 5 days of symptom onset also reduces the risk of Long Covid by 26% at 6 months after treatment initiation (28). Nirmatrelvir-ritonavir is administered orally. In accordance with its Summary of Product Characteristics (SmPC) and the potential risks of drug interactions, special caution should be taken before administrating nirmatrelvir-ritonavir to patients who are already taking medications (29). In patients treated with nirmatrelvir-ritonavir, the duration of illness is shorter and viral load is decreased (30, 31). COVID-19 rebound have been observed in some patients (30). However, there is currently only mixed evidence of a link between antiviral treatment and a rebound effect: rebound might be a natural phenomenon unrelated to antiviral treatment (30, 31). Data on larger population will need to be further investigated.

Since October 2023, Veklury® (remdesivir) has also been recommended for the treatment of COVID-19 in the same eligible population as those targeted by nirmatrelvir-ritonavir. An observational study showed a 59% reduction in hospitalizations and emergency admissions with the administration of remdesivir in this target population (OR = 0.41, 95% CI = 0.17–0.95) (32). However, its intravenous formulation requires administration in a hospital setting. While both nirmatrelvir-ritonavir and remdesivir are recommended by the Belgian healthcare system for similar population, the eligible patients might differ in clinical practice due to the specific features of each antiviral. There is no direct comparison between both antiviral treatments.

The primary objective of this paper is to investigate the cost-effectiveness of nirmatrelvir-ritonavir compared to no anti-SARS-CoV-2 treatment in persons at high risk of progressing to severe COVID-19. This analysis is from the healthcare payer’s perspective and in the Belgian setting, considering the Omicron variant of SARS-CoV-2. For comprehensive information, the cost-effectiveness of remdesivir compared to no anti-SARS-CoV-2 treatment is also presented. Remdesivir is expected to be administered in cases where nirmatrelvir-ritonavir is contraindicated.

The transmissibility and severity of SARS-CoV-2 have evolved over time. The emergence of new variants or coronaviruses strains is likely. The added value of our study is to estimate the cost-effectiveness of nirmatrelvir-ritonavir using effectiveness data from real-world settings and accounting for the impact of Omicron-SARS-CoV-2 infections on hospitalizations and deaths in 2022.

## Materials and methods

This cost-effectiveness analysis is based on a static model. Modeling transmission of the SARS-CoV-2 virus is complex and highly uncertain as known variants of this virus are characterized by different transmission patterns and disease severity. The choice for a static model is justified by the reference to well-documented epidemiological data in the total population over the study period. However, it disregards the reduction in transmission risk due to effective antiviral treatment. The cost-effectiveness of nirmatrelvir-ritonavir has been studied during the one-year Omicron period (2022), with easy access to vaccination. During this period, the Belgian Health Institute established a performant infrastructure reporting daily cases by age groups as well as the number of hospitalizations, ICU admissions and deaths related to SARS-CoV-2 virus. The effectiveness of nirmatrelvir-ritonavir has been investigated in large real-world settings (33). The present analysis does not capture the benefit of a shorter symptomatic period on the risk reduction of virus transmission to other subjects.

This analysis targets the population aged 65 years and older infected by SARS-CoV-2 virus. It is assumed that the impact of SARS-CoV-2 on this population’s health is a good proxy of its impact on other frail sub-populations, especially if they have not been vaccinated against SARS-CoV-2. The Belgian healthcare system recommends nirmatrelvir-ritonavir for these sub-populations, which include patients aged 65 years or older with at least one specific underlying chronic health condition, patients with severe immunosuppression and patients with heart failure or COPD (chronic obstructive pulmonary disease) (34). The absence of a specific anti-SARS-CoV-2 treatment was used as comparator of this analysis. Nirmatrelvir-ritonavir was also compared to placebo in the pivotal clinical trial with high-risk patients (25). Compared to placebo, remdesivir also showed effectiveness in this target population. For completeness purpose, the cost-effectiveness of this antiviral compared to no anti-SARS-CoV-2 treatment will be included in this analysis. Remdesivir is expected to be administered in cases where nirmatrelvir-ritonavir is contraindicated.

The results have been presented for a treated patient cohort. A patient cohort-based approach was chosen because we are currently experiencing an endemic situation. In a pandemic situation, a broader population-based approach, considering the results within a total (national) population where antiviral intervention is only used in a defined share of eligible patients, would have been more appropriate for healthcare decision making. Furthermore, a cohort-based approach is more common and easy to interpret (35).

### Model overview

A decision tree has been developed to capture the number of days with symptoms, the number of hospital admission, post-acute COVID-19 syndromes (PACS; also known as Long Covid) and deaths in a hypothetical cohort of 1,000 high-risk infected patients. Decision trees have previously been used in studies evaluating health economic outcomes for respiratory illnesses, including COVID-19 (36).

The decision tree has been split into two sub-decision trees (hospitalized and ambulatory settings) to further describe the health evolution of symptomatic patients at high risk of severe disease. This decision has been presented in Carlson et al. publication (37).

The ambulatory sub-decision tree calculates the costs and quality of life for patients managed in ambulatory settings. It is assumed that these patients, who do not require hospitalization, present mild to moderate symptoms and only require rest before recovery. All these patients are assumed to survive.

The hospitalized sub-decision tree assesses the costs and mean number of days in general wards and intensive care units (with or without mechanical ventilation). In-hospital treatment with antiviral agents (or monoclonal antibodies) is not considered. Each hospital stay is associated with a mortality risk.

In case of survival, both symptomatic hospitalized and ambulatory patients may suffer from long-term PACS or may be fully cured.

The decision tree does not consider adverse events related to the intervention. Severe adverse events were similar in control and treated patient groups within the pivotal clinical trial of nirmatrelvir-ritonavir (25).

Direct healthcare costs are evaluated by multiplying the case-specific counts (outpatient cases, hospitalizations and PACS) with the mean unit case-specific cost. Baseline utilities are derived from the general population values and adjusted by disutilities associated with COVID-19 symptom days, hospitalization (in general ward, ICU with or without mechanical ventilation) and PACS (12, 38, 39).

The costs and consequences of COVID-19 infection are evaluated using a daily model cycle, reflecting disease dynamics. A one-year time horizon has been applied for the costs related to COVID-19 infection, including the cost for Long Covid, limited to 1 year in the absence of long-term robust data. The effect on death has been observed with nirmatrelvir-ritonavir treatment. The lifetime impact of prevented premature death was considered in this cost-effectiveness analysis.

### Inputs

#### Target population

The target population of this cost-effectiveness analysis comprises infected patients aged 65 years and older. According to the National Health Institute Sciensano, 79.01% of these patients were vaccinated in 2022. Individuals who had not received a booster or vaccination over the last 6 months were considered unvaccinated.

#### Hospitalization and death

During the first year of Omicron variant circulation (2022), 12.2% of the target population were hospitalized. Data on hospitalization admission are reported by a Belgian representative hospital network (4, 9). Based on this aggregated dataset, we calculated that 4.9% of the hospitalized target population was admitted to the intensive care unit, with 16.1% of these requiring mechanical ventilation. A higher hospitalization rate was reported in the unvaccinated population (19.7% vs. 10.2% in the vaccinated group).

From this Belgian aggregated dataset, it was estimated that 6.4% of the target population died from SARS-CoV-2 infection in hospitals in 2022. A slightly higher mortality rate was observed in the unvaccinated population (6.7% vs. 6.3% in the vaccinated group).

#### Long Covid

Patients infected with SARS-CoV-2 are at risk of developing Long Covid. Studies, including the COVIMPACT survey conducted by the Belgian Health Institute Sciensano, have highlighted that hospitalized patients were at higher risk of Long Covid (11, 12, 40). Preventing hospitalization will consequently reduce the risk of Long Covid. The COVIMPACT study reports that, on average, 41.6% of infected patients who required hospitalization still suffer from at least one symptom of COVID-19 disease six months after infection diagnosis and did not feel recovered. This rate is 60% higher than the rate observed in infected patients who recovered at home (26.0%). However, there is no specific data for the target population of the present analysis (65 years and older). Data from the COVIMPACT study at 12 months have not been disclosed. The six-month rate has been applied as a mid-point over the one-year time horizon. This parameter will be subject to scenario analysis.

#### Utilities

SARS-CoV-2 infection impacts patients’ quality of life. A baseline utility is assigned at the start of the infection episode: the mean EQ-5D-5L utility value in the Belgian general population aged 65 years and older is estimated at 0.77 (38). The infected patient progresses through different health events in the decision tree (infection with mild/moderate symptoms, hospitalization, admission to ICU, occurrence of Long Covid) before recovery or death. The disutilities associated with these health events are reported in Table 1. Except for the disutility associated with Long Covid, these values were derived from non-Belgian literature on other respiratory infections.

**Table 1:** Input values of the cost-effectiveness analysis.

Parameters	Mean Values	Source
Disutilities		
Disutility during Covid-19 symptomatic infection days (mild to moderate symptom)	−0.290	(39)
Disutility during hospitalization (severe symptom)	−0.640	(38)
Disutility during hospitalization in ICU (without mechanical ventilation)	−0.570	(38)
Disutility during hospitalization in ICU (with mechanical ventilation)	−0.770	(38)
Disutility with Long Covid complication	−0.121	(12)
Effectiveness of nirmatrelvir+ritonavir		
Reduction of hospitalizations	79.6%	(26)
Reduction in deaths	79.6%	(26)
Reduction in symptomatic days	20.0%	(25)
Effectiveness of remdesivir		
Reduction in hospitalization	59.0%	(32)
Reduction in death	59.0%	Assumption (same as for hospitalization)
Estimated costs (€ 2023)		
Treatment cost with nirmatrelvir-ritonavir (oral formulation)	€957.85	www.inami.fgov.be; pack of 30 tablets. Presuming 50% delivered by public pharmacy and 50% by hospital pharmacy
Treatment cost with remdesivir (IV formulation)	€ 1,637.5 for drug + €208.4 for IV administration	www.inami.fgov.be; 4 vials remdesivir and 3 days of day clinic admission
Cost of treatment initiation	€30.00	Presuming a general practitioner visit
Cost of Hospital admission due to severe COVID-19 infection	€10,802	Cost per day: NIHDI (year 2020 inflated to year 2023) Mean number of days: Sciensano
Estimated mean annual cost to manage Long Covid complication	€ 360	Frequency of key Long Covid symptoms: 10% anxiety, 12% depression and 60% pain (12). Unit cost of anxiety and depression: (45) Unit cost of Long Covid care scheme: www.inami.fgov.be
Estimated mean cost in the first year after hospital discharge	€ 814	Frequency of healthcare resources: (47, 48), adjusted (exclusion of rehabilitation cost due to lower ICU admission in Omicron period) and data from one hospital to estimate the risk of re-hospitalization.

ICU, intensive care unit.

#### Effectiveness

The effectiveness of nirmatrelvir+ritonavir is defined by the reduction in the number of symptomatic days, hospitalizations, deaths and Long Covid cases. The reduction in hospitalizations and deaths is derived from an observational study (26). The effect on Long Covid is indirectly inferred from the decrease in hospitalization stays and the duration of COVID-19 disease in ambulatory setting. Recent findings by Xie et al. confirm the reduction of Long covid cases in patients treated with nirmatrelvir+ritonavir (28). The reduction in virus transmission due to a shorter duration of illness has not been considered in the analysis.

For completeness, the antiviral remdesivir has also been included in the comparison with no anti-SARS-CoV-2 treatment. The effectiveness of remdesivir is derived from an observational study (32). High-risk outpatients with Omicron-related COVID-19 were significantly less likely to be hospitalized or visit the emergency department within 29 days from symptom onset compared to a control cohort who did not receive therapy (OR = 0.41, 95% CI = 0.17–0.95). No conclusions regarding mortality could be drawn from this study. We have assumed that remdesivir has a similar effectiveness on death as it does on hospitalization.

To estimate the impact on ICU admissions, we refer to the Belgian Health Institute data on the number of ICU admissions among the target patients hospitalized during the first year of Omicron variant (2022).

The effectiveness data are summarized in Table 1. Effectiveness outcomes will be further investigated in the sensitivity analyses.

#### Costs

Direct healthcare costs associated with COVID-19 disease include the cost of antiviral treatment and the direct healthcare resources used by patients treated at home or in hospital. Survivors may also incur direct healthcare costs related to Long Covid. Ambulatory costs associated with drugs aimed at alleviating infection symptoms (e.g., fever, cough) have not been included in this study as these costs are minor and expected to be similar in both intervention arms. Healthcare costs associated with antiviral treatment adverse events have also been disregarded as they are expected to be minor if the drugs are prescribed according to the label (24). Due to mixed evidence of a link between antiviral treatment and rebound effect, no costs associated with a potential rebound effect have been considered in this analysis.

In line with the Belgian health economic guidelines, we have adopted the healthcare payer’s perspective, including public payer and patient (41). Unit costs were inflated to year 2023 based on the health index, if necessary.

It is assumed that all high-risk patients infected with SARS-CoV-2 will visit a physician.

Patients treated with nirmatrelvir-ritonavir will not require additional visits. Patients treated with remdesivir will receive the antiviral in a one-day clinic over 3 days, at an administration cost of €69.47 per day.

The mean cost for hospitalization is estimated at €10,802 based on the length of stay reported by the Belgian Health Institute in 2022 and the unit cost per hospitalization day with COVID-19 infection, as estimated by the National Institute for Health and Disability Insurance (NIHDI) and the hospital daily cost for the patient. Data from 2023 confirm the stability of the length of stay in the older adult population hospitalized for COVID-19 infection (42).The mean estimated hospitalization cost is also in line with recent Belgian cost reported for patients aged 65 years and older hospitalized for other respiratory diseases (43–45).

To our knowledge, no data are available on the cost of Long Covid in the Belgian context. This cost was estimated based on the probabilities of symptoms associated with Long Covid and derived from the COVIMPACT survey (12) and healthcare cost unit for management of these symptoms as reported in literature for anxiety and depression (46), the pharmaceutical specialties database^1^ for pain and NIHDI portal^2^ for the Long Covid Care scheme. The latest available results of the COVIMPACT study report 10% of additional patients suffering from anxiety after 6 months since infection (compared to the situation at the time of infection), and 12% of additional patients suffering from depression (12). Sixty percent of the patients with Long Covid also reported experiencing pain (in the head or muscles) (12). Based on the budget allocated by NIHDI for the Long Covid care scheme and the incidence of SARS-CoV-2 infections, it was estimated that 2% of the patients suffering from Long Covid would be included in this specific care scheme. Only direct healthcare costs associated with Long Covid were considered in this analysis.

After hospital discharge, the patients severely affected by this respiratory infection will need close follow-up. This monitoring has been described in two Belgian publications and includes physician visits (31.8%), laboratory tests (31.8%), pulmonary function testing (31.8%), functional assessment (31.8%), CT chest imaging (15.1%), transthoracic electrocardiography (27.5%), fibrosis assessment (10.0%) and, exceptionally, re-hospitalization (3.8%) (47, 48). This close follow-up is especially relevant for patients who have been admitted to the ICU (49). These healthcare resources have been valued with unit cost as reported in the NIHDI database (10). The data have been reviewed during two advisory board meetings with Belgian physicians. The aggregated cost inputs are summarized in Table 1.

### Base case analysis

The cost-effectiveness results of nirmatrelvir-ritonavir and remdesivir are expressed in incremental costs, QALYs and the ratio of these parameters (ICER—Incremental Cost-Effectiveness Ratio).

### Sensitivity analysis

The sensitivity analyses are only conducted for the reference case of nirmatrelvir-ritonavir compared to no anti-SARS-CoV-2 treatment. The results of the one-way deterministic sensitivity analysis are reported in a Tornado diagram. The values of the key parameters were adjusted by +/−20% around the mean (in absence of 95%CI data).

The probabilistic sensitivity analysis is based on 1,000 simulations. In each simulation, all relevant inputs were randomly drawn from pre-specified distributions to inform the possible range of values. A beta distribution was applied to parameters that needed to remain bounded between 0 and 1 (such as proportions, utilities and disutilities) and a standard gamma distribution to the cost parameters. The results are reported in a cost-effectiveness plane and a cost-effectiveness acceptability curve to graphically illustrate the level of variability and uncertainty in the results.

### Scenario analysis

In addition to the deterministic and probabilistic sensitivity analyses, two additional scenarios were explored. The first scenario tested the extreme situation of nirmatrelvir/ritonavir having no impact on Long Covid. The second scenario analyzed the impact of a lower vaccination rate in the target population.

## Results

In Table 2, we present the cost-effectiveness results for a hypothetical cohort of 1,000 Belgian patients aged 65 years and older who are at risk of severe COVID-19 disease.

**Table 2:** Cost-effectiveness results over lifetime horizon for a cohort of 1,000 infected patients at high risk of severe COVID-19 disease.

For a cohort of 1,000 infected patients at high risk of severe COVID-19 disease	Treatment with nirmatrelvir-ritonavir	No specific anti-SARS-CoV-2 treatment	Treatment with remdesivir	Difference nirmatrelvir-ritonavir vs. no specific anti-SARS-CoV-2 treatment	Difference remdesivir vs. no specific anti-SARS-CoV-2 treatment
Effects					
Hospitalizations (n)	25	122	50	−97	−72
ICU admissions (n)	1	6	2	−5	−4
Deaths (n)	0	8	1	−8	−7
Long Covid complications (n)	263	273	266	−10	−7
QALYs (n)	12,946	12,849	12,932	97	83
Healthcare costs					
Costs related to GP visit	€30,000	€30,000	€30,000	€0	€0
Costs due to hospitalization	€265,780	€1,302,844	€534,166	−€1,037,064	−768,678
Costs to manage Long Covid (1 year)	€94,632	€98,076	€95,658	−€3,443	−€2,418
COVID-19 specific antiviral treatment	€957,850	€0	€1,845,930	€957,850	€1,845,930
Total costs	€1,348,262	€1,430,920	€2,505,754	−€82,658	€1,074,834

Over a lifetime horizon, nirmatrelvir/ritonavir provides 97.5 additional QALYs to the treated cohort, compared to the untreated cohort. Moreover, patients treated with nirmatrelvir/ritonavir benefited from a lower risk of hospitalization, resulting in savings in hospitalization costs and costs associated with Long Covid complications. These healthcare savings exceed the acquisition costs of nirmatrelvir/ritonavir. Treating 1,000 high risk patients with nirmatrelvir/ritonavir saves €82,658 in the healthcare system.

Treatment with remdesivir provides 83.5 additional QALYs compared to no anti-SARS-CoV-2 treatment but increases healthcare costs by €1,074,834 for the treatment of 1,000 patients aged 65 years and older at risk of severe COVID-19 disease.

**Figure 1:**
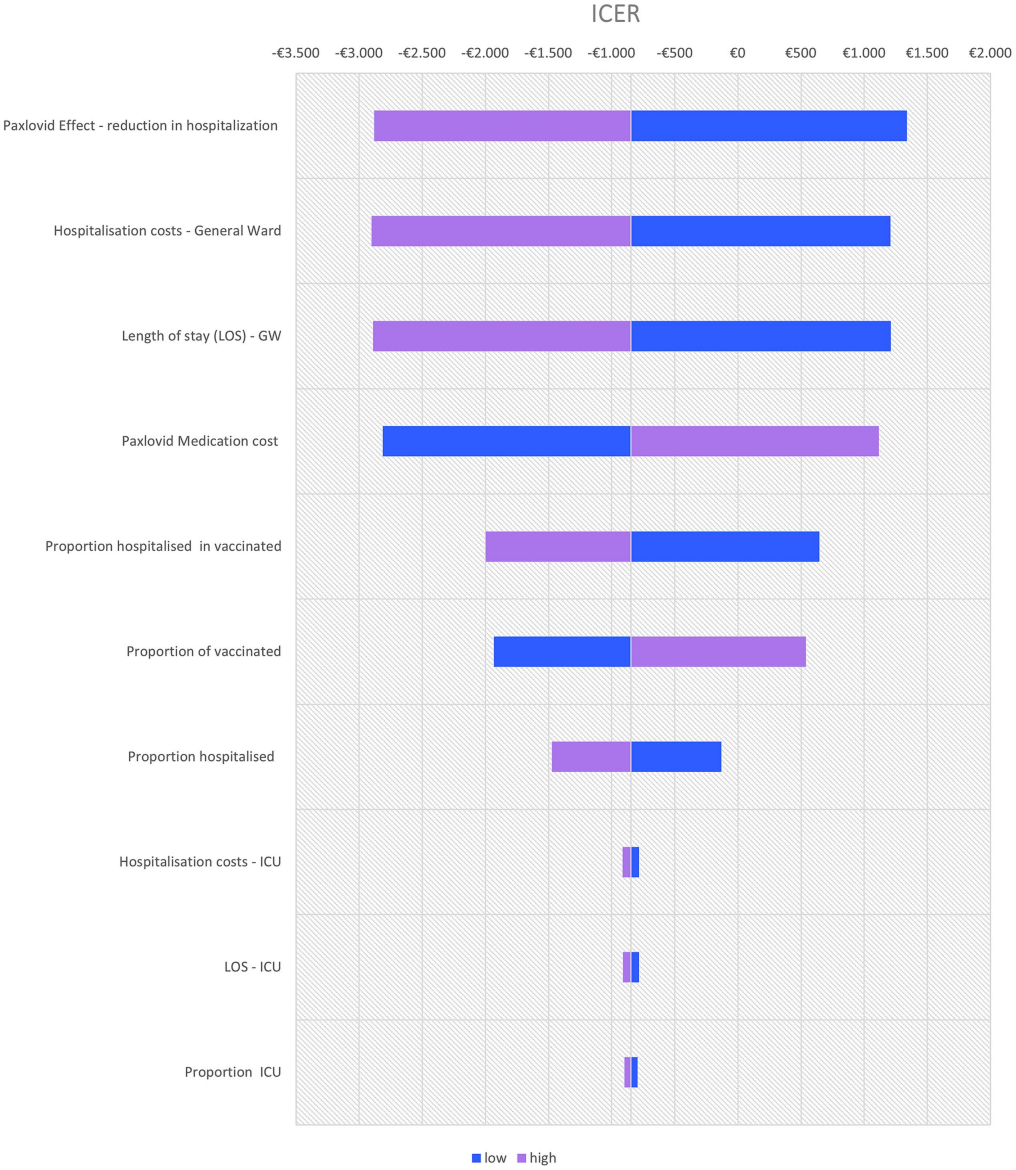
Tornado diagram of key drivers of cost-effectiveness of nirmatrelvir-ritonavir vs. no anti-SARS-CoV-2 treatment (variation with +20% and –20% of the mean value used in base case).

A deterministic sensitivity analysis identified the following four key drivers of the cost-effectiveness of nirmatrelvir/ritonavir: the effectiveness of nirmatrelvir-ritonavir in reducing hospitalization, the hospitalization cost, the length of hospital stays, and the cost of nirmatrelvir-ritonavir (Figure 1).

Even if Long Covid is excluded from the analysis, nirmatrelvir-ritonavir remains a cost-saving option, with €79,214 in savings for a cohort of 1,000 high-risk patients. If the vaccination rate among individuals older than 65 years is lower (e.g., 55%), the savings would increase to €278,123 and the number of QALYs to 115.6, making nirmatrelvir-ritonavir even more cost-saving.

**Figure 2:**
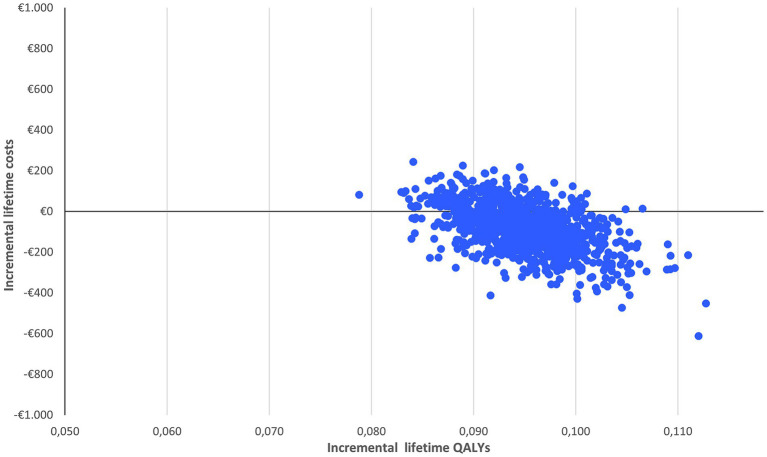
Probabilistic sensitivity analysis of nirmatrelvir-ritonavir treatment vs. no anti-SARS-CoV-2 treatment.

**Figure 3:**
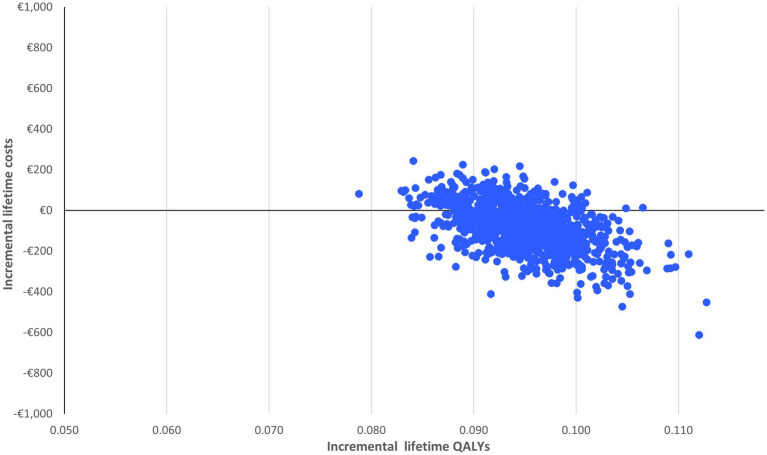
Cost-effectiveness acceptability curve of nirmatrelvir-ritonavir treatment vs. no anti-SARS-CoV-2 treatment.

With limited spread of the simulations in the cost-effectiveness plane (Figure 2), the probabilistic sensitivity analysis confirms the robustness of the results: nirmatrelvir-ritonavir is a cost-saving option in 75% of the cases and has a 100% probability of being cost-effective at a willingness to pay of €2,000/QALY (Figure 3).

## Discussion

Our cost-effectiveness analysis, based on a decision tree and real-world data, has demonstrated the cost-saving nature of nirmatrelvir-ritonavir as compared to no anti-SARS-CoV-2 treatment in the Belgian setting with the Omicron variant SARS-CoV-2 in patients at high risk of hospitalization. Extensive sensitivity analyses have corroborated this conclusion: in 75% of the simulations, nirmatrelvir-ritonavir is a cost-saving option in individuals aged 65 years and older, considered as a good proxy of patients at high risk of hospitalization. In 100% of the cases, it is cost-effective at a willingness to pay of €2,000/QALY. The deterministic sensitivity analysis considering +/−20% variation around the mean value of key parameters (including effectiveness) also supports the cost-effectiveness outcomes. Compared to no anti-SARS-CoV-2 treatment, the alternative antiviral treatment remdesivir is never cost-saving nor cost-effective.

In the current endemic situation with the SARS-CoV-2 virus, the COVID-19 vaccination rate in the Belgian population aged 65 years and older is lower (42) compared to the vaccination rate in the reference year of this cost-effectiveness analysis (2022), making the target population more at risk of infection and hospitalization. Similar vaccination uptake is observed in the high-risk group of immunocompromised patients. Additionally, individuals older than 65 years old still represent the majority of hospitalized patients with COVID-19 (50). These hospitalized patients usually present with one or more co-morbidities. In the endemic context, the number of hospitalizations due to COVID-19 in the older adult population is as relevant as the number of hospitalizations due to other severe respiratory diseases such as influenza (42). An antiviral treatment that prevents severe COVID-19 infection should be adequately administered to patients at higher risk of severe symptoms to prevent their hospitalization and associated complications.

The major limitation of our study relates to the consistent effectiveness of the antiviral in all high-risk patients against the evolving features of the infection and changing immunity patterns of the patients. The effectiveness outcomes derived from a US observational study have been considered the best available data to apply in this Belgian analysis (26). Such analysis should be re-iterated in the future with updated data.

The cost and length of hospital stay also influence the cost-effectiveness results. The hospitalization data due to COVID-19 infection in 2023 supports the length of hospital stay and related healthcare costs used in the present analysis. Only conservative healthcare cost has been considered for Long Covid, disregarding the potential high impact of Long Covid on patient’s productivity loss. To our knowledge, no published study has yet provided data on the healthcare cost of Long Covid in Belgium. Consequently, this cost was derived from conservative estimates. The scenario analysis has tested this parameter. Due to lack of data, the disutility related to Long Covid has also been derived from data on other respiratory infections (39).

Carlson et al. have recently published a cost-effectiveness analysis of nirmatrelvir-ritonavir for a younger population (45 years) at high risk of progression to severe COVID-19 in the United States (US) (37). In this population, they concluded that nirmatrelvir-ritonavir compared to no anti-SARS-CoV-2 treatment, was a very cost-effective option. The economic analysis reported by the Institute for Clinical and Economic Review (ICER) in the United States was referring to a broader patient population with far lower risk of hospitalization (0.96%) than the one observed in Belgium for the specific target patients that are enrolled in this Belgian cost-effectiveness analysis (51).

We have adopted the standard narrow healthcare payer perspective in terms of the scope of benefits included in an economic evaluation. This standard approach might be complemented with the inclusion of broader benefits associated with this effective antiviral treatment such as the ‘insurance value’, defined as the availability of a treatment in case of a crisis situation with limited ICU capacity (52). This broader perspective is not part of the present analysis as we lack validated methodology to include this value but might be included in future research (53–56).

## Conclusion

When administered in line with its EMA-approved label, specifically to patients at high risk of progression to severe COVID-19, this study shows that nirmatrelvir-ritonavir not only saves health care costs related to hospitalization and Long Covid complications, but also generates health benefits. It reduces the risk of premature death associated with COVID-19 and prevents the quality of life loss due to hospitalization, in the Belgian setting with the Omicron-variant SARS-CoV-2.

## Data Availability

The datasets presented in this study can be found in online repositories. The names of the repository/repositories and accession number(s) can be found in the respective references or footnotes.

## Footnotes

^1^ https://www.cbip.be/

^2^ https://webappsa.riziv-inami.fgov.be/Nomen/fr/search
